# Kaempferol Ameliorates the Inhibitory Activity of Dexamethasone in the Osteogenesis of MC3T3-E1 Cells by JNK and p38-MAPK Pathways

**DOI:** 10.3389/fphar.2021.739326

**Published:** 2021-10-05

**Authors:** Baocheng Xie, Zhanwei Zeng, Shiyi Liao, Chenhui Zhou, Longhuo Wu, Daohua Xu

**Affiliations:** ^1^ Guangdong Key Laboratory for Research and Development of Natural Drugs, The Public Service Platform of South China Sea for R&D Marine Biomedicine Resources, Marine Biomedical Research Institute, Guangdong Medical University, Zhanjiang, China; ^2^ Department of Pharmacy, Affiliated Dongguan Hospital, Southern Medical University, Dongguan, China; ^3^ Key Laboratory of Traditional Chinese Medicine and New Pharmacy Development, Guangdong Medical University, Dongguan, China; ^4^ School of Nursing, Guangdong Medical University, Dongguan, China; ^5^ College of Pharmacy, Gannan Medical University, Ganzhou, China

**Keywords:** kaempferol, osteogenic differentiation, dexamethasone, osteoporosis, JNK and p38 MAPK signaling pathways

## Abstract

Kaempferol has been reported to exhibit beneficial effect on the osteogenic differentiation in mesenchymal stem cells (MSC) and osteoblasts. In our previous study, dexamethasone (DEX) demonstrated inhibitory effect on MC3T3-E1 cells differentiation. In this study, we mainly explored the protective effect of kaempferol on the inhibitory activity of DEX in the osteogenesis of MC3T3-E1 cells. We found that kaempferol ameliorated the proliferation inhibition, cell cycle arrest, and cell apoptosis and increased the activity of alkaline phosphatase (ALP) and the mineralization in DEX-treated MC3T3-E1 cells. Kaempferol also significantly enhanced the expression of osterix (Osx) and runt-related transcription factor 2 (Runx2) in MC3T3-E1 cells treated with DEX. In addition, kaempferol attenuated DEX-induced reduction of cyclin D1 and Bcl-2 expression and elevation of p53 and Bax expression. Kaempferol also activated JNK and p38-MAPK pathways in DEX-treated MC3T3-E1 cells. Furthermore, kaempferol improved bone mineralization in DEX-induced bone damage in a zebrafish larvae model. These data suggested that kaempferol ameliorated the inhibitory activity of DEX in the osteogenesis of MC3T3-E1 cells by activating JNK and p38-MAPK signaling pathways. Kaempferol exhibited great potentials in developing new drugs for treating glucocorticoid-induced osteoporosis.

## Introduction

Glucocorticoids are widely used in the anti-inflammatory treatment of various immune-mediated diseases, such as rheumatic arthritis and inflammatory bowel disease (Güler-Yüksel et al., 2018; Vandewalle et al., 2018). However, long-term use of glucocorticoids could cause a variety of serious adverse effects. Glucocorticoids inhibited cellular proliferation, osteoblast differentiation, and mineralization of osteoblasts and induced cell apoptosis *in vivo* and *in vitro* (Yoon et al., 2012; Chen et al., 2016). Glucocorticoid-induced osteoporosis (GIOP), a secondary iatrogenic osteoporosis, is one of the most common clinical complications (Buehring et al., 2013). A research indicated that 30–50% patients with GIOP would have the risk of fractures if they were treated with long-term glucocorticoids and that the risk would never return to the baseline after discontinuing administration of glucocorticoids (Buehring et al., 2013). Fracture is a significant cause of increased mortality and reduced quality of life. Therefore, it is critical to discover new drugs to counteract GIOP.

Kaempferol, a kind of flavonol and polyphenol antioxidant, has been found in many plants, including tea, apples, and beans and used for treating tumors and inflammatory diseases (Chen and Chen, 2013; Song et al., 2015; Molitorisova et al., 2021; Wang et al., 2021). Studies also demonstrated that kaempferol could stimulate osteogenic differentiation of mesenchymal stem cells (MSC) and osteoblasts (Yang et al., 2010; Byun et al., 2012; Kim et al., 2016; Adhikary et al., 2018). However, the mechanism by which kaempferol prevents dexamethasone-induced osteoporosis remains unclear.

Mitogen-activated protein kinases (MAPK) pathway is one of the most crucial pathways in osteoblast differentiation, and regulated by their phosphorylation levels (Greenblatt et al., 2013; Wang et al., 2018). It has been shown that the flavonoid compound puerarin promotes the activity of osteogenic differentiation in MSC, which is mediated by MAPK signaling pathways (Yang et al., 2018). Our previous researches also showed that amentoflavone and apigenin improved the osteogenesis of human MSC by regulating JNK and p38-MAPK pathways (Zhang et al., 2015; Zha et al., 2016).

In this study, we explored the roles of kaempferol on the suppression of osteogenesis induced by dexamethasone (DEX) in MC3T3-E1 cells and zebrafish. The experimental results revealed that kaempferol improved osteogenesis that was suppressed by DEX. The mechanisms might be associated with activation of JNK and p38-MAPK pathways. These results have revealed that kaempferol is potentially a useful candidate drug for the treatment of GIOP.

## Materials and Methods

### Cell Culture

MC3T3-E1 cells was cultured in α-MEM (A1049001, Gibco) supplemented with 10% (v/v) fetal bovine serum (FBS) (10099-141, Gibco) solution and were incubated at 37°C in 5% CO_2_ humidified air.

### Solution Preparation

Kaempferol was dissolved in dimethyl sulfoxide (DMSO) at 100 mM and kept away from light at −20°C for use, which was diluted with α-MEM to a suitable concentration upon experiment.

### Cell Viability and Proliferation Assay

Cell counting kit-8 (CCK-8) assays were employed to assess the cell viability, according to the instructions of manufacture (Dojindo laboratories, Japan). Specifically, MC3T3-E1 cells were grown in 96-well plates (3 × 10^3^ cells/well). To assess the effect of kaempferol on the cell viability of osteoblasts, cells were co-cultured with 1, 5, 10, 25, 50, 75, and 100 μM kaempferol, respectively. To evaluate the protective activity of kaempferol in DEX-induced osteoblast viability, cells in experimental groups were co-cultured with 5, 10, and 25 μM kaempferol, respectively, and 1 μM DEX for 3 days. CCK-8 (10 μl) with complete medium (90 μl) was then added, and cells were cultured in carbon dioxide (CO_2_) incubator for an additional 2 h. The wavelength 450 nm was set for the absorbance, and the assays were conducted on a microplate reader (Thermo Fisher Scientific Inc., United States). MC3T3-E1 cells were seeded in 24-well plates for 48 h and cell proliferation was evaluated using the BeyoClick™ EdU-594 (C0078S, Beyotime). Briefly, EdU (10 µM) was added into the cell culture medium and incubated for 2 h. The cells were then treated with 4% paraformaldehyde for 15 min and 0.5% Triton X-100 for 10 min. After washing with PBS, the cells were stained at room temperature for 30 min with the Click Additive Solution. Next, the cells were incubated with Hoechst 33342 stain for 10 min. Finally, a fluorescence microscope (Olympus Corporation) was used to observe and count the percentage of EdU+ cells from 4 optical fields/well.

### Cell Cycle Analysis

Cell cycle was measured by flow cytometry. Briefly, digested cells were harvested. Cell pellet was suspended with 70% ethanol at 4°C overnight. The stationary cells were co-incubated with RNase-A and propidium iodide (PI) for half an hour. Cell cycle was analyzed by FACSCalibur (BD Biosciences Pharmingen™).

### Apoptotic Assay

The cells were fixed with 4% paraformaldehyde, then stained with Hoechst 33342 (C1026, Beyotime) for 20 min and washed with PBS for 3 times. Apoptosis of the cells was observed under fluorescence microscope (Olympus Corporation). Cells were stained with PI and annexin V-FITC to evaluate apoptosis by flow cytometry according to the manufacturer’s instructions (BD Biosciences PharMingen). Cells were washed twice with phosphate-buffered saline (PBS) and stained with 5 μl of Annexin V-FITC and 5 μl of PI for 15 min in the dark. Quantification of apoptotic cells was performed by flow cytometry using a FACScan cytofluorometer (BD Biosciences).

### ALP Activity and Staining Assay

To explore the roles of kaempferol in osteogenic differentiation, cells in experimental groups were treated with 5, 10, and 25 μM kaempferol, respectively, and 1 μM dexamethasone. The ALP activity was measured by an ALP Assay Kit after 5 days. The wavelength of 405 nm was set for detection in microplate reader. ALP staining was conducted by BCIP/NBT Kit (C3206, Beyotime).

### Mineralization Assay

Mineralization activity was investigated by alizarin red staining. MC3T3-E1 cells (2×10^5^ cells/well) were induced by osteogenic induction medium (OIM, including 50 μg/ml ascorbic acid, 10 mM β-glycerophosphate). Cells in experimental groups were treated with 5, 10, and 25 μM kaempferol, respectively, and 1 μM DEX for 14 days. Then, MC3T3-E1 cells were stained using alizarin red S for half an hour, and the images of mineralization nodes were photographed. 10% cetylpyridinium chloride (CPC, Sigma) was used to extract the alizarin red, and the wavelength 562 nm was selected for detection.

### Gene Expression Analysis

TRIZOL reagent was employed for extracting total RNA, which was reversely transcribed into the cDNA, according the instructions of a DNA Reverse Transcription Kit. Reverse transcription reaction conditions: Ⅰ, 37°C, 15 min; Ⅱ, 85°C, 5 s; Ⅲ, 4°C storage. The cDNA was synthesized using a Prime Script TMRT reagent Kit with gDNA Eraser (TaKaRa). The qRT-PCR was analyzed by SYBR Premix Ex TaqII Reverse Transcriptase (TaKaRa). All primers used in this study were listed as below: Runx2 forward: 5′-gaa​tgc​act​acc​cag​cca​c-3′, reverse: 5′-tgg​cag​gta​cgt​gtg​gta​g-3′; Osx forward: 5′-agg​agg​cac​aaa​gaa​gcc​ata​c-3′, reverse: 5′-agg​gaa​ggg​tgg​gta​gtc​att-3′; β-actin forward: 5′-gcc​aac​cgt​gaa​aag​atg​ac-3′, reverse: 5′-acc​aga​ggc​ata​cag​gga​cag-3′. Amplification conditions: Ⅰ. Pre-denaturation: 95°C, 30 s; Ⅱ. Amplification: 95°C, 5 s, 60°C, 34 s, 40 cycles; Ⅲ. Dissolution curve: 95°C, 30 s, 65°C, 30 s, 97°C, 30 s; Ⅳ. Store at 4°C for gene amplification. In this study, 7,500 real-time quantitative fluorescence PCR system was used for real-time quantitative PCR analysis, and all the sample were normalized to β-actin.

### Western Blot Analysis

The extracts of total protein were prepared by lysing MC3T3-E1 cells in a RIPA buffer containing protease inhibitors. BCA assays were employed to determine the concentrations of protein. 30 μg proteins of each sample were added to 10% SDS-PAGE and then electrotransferred to the PVDF for further immunoblotting. Antibodies were used: anti-Runx2 (1:1000, ab236639, Abcam), anti-Osx (1:1000, ab209484, Abcam), anti-p53 (1:1000, 60283-2-Ig, Proteintech), anti-CyclinD1 (1:1000, 26939-1-AP, Proteintech), anti-Bcl-2 (1:1000, 26593-1-AP, Proteintech), anti-Bax (1:1000, 60267-1-Ig, Proteintech), anti-p38 (1:1000, #8690, Cell Signal Technology), anti-p-p38 (1:1000, #4511, Cell Signal Technology), anti-JNK (1:1000, #9255, Cell Signal Technology) anti-p-JNK (1:1000, #9255, Cell Signal Technology), and anti-β-actin antibody (1:1000, AF0003, Beyotime). PVDF were incubated with diluted antibodies. Goat anti-rabbit IgG H&L (HRP) (1:5000, ab6721, Abcam) or goat anti-mouse IgG H&L (HRP) (1:5000,ab6789, Abcam) was used to co-incubate with PVDF at 37°C for 1 h. Proteins were detected by ECL, and quantitatively analyzed by scanning densitometry (Bio-Rad, United States).

### Alizarin Red Staining and Quantitative Analysis of Mineralization

Wild type zebrafish larvae (AB strain) were purchased from Shanghai FishBio Co., Ltd. Larvae of AB strain were cultured in medium (0.16 mmol/L MgSO_4_, 0.33 mmol/L CaCl_2_, 0.17 mmol/L KCl, 5 mmol/L NaCl, and 10 ppm methylene blue) under isothermal conditions at 28.5°C. On 9 dpf (days post-fertilization), the zebrafish larvae were stained with alizarin red. First, zebrafish larvae were immobilized in 4% polyformaldehyde for 2.5 h and dehydrated in 50% ethanol for 20 min. Then, zebrafish larvae were bleached with 1.5% H_2_O_2_ in 1% KOH for 25 min to remove the pigment and washed 3 times with PBST. Next, zebrafish larvae were stained with 0.01% alizarin red staining in 0.7% KOH for 4 h. Finally, the zebrafish larvae were decolorized in different proportions of 0.5% KOH and glycerin (3:1, 1:1, 1:3) for 6–8 h. The experimental protocols for quantifying larval skull mineralization were similar to those previously published (Wu et al., 2021). Zebrafish were placed on a slide covered with glycerin. Stereomicroscope was used to photograph ventral view and lateral view of zebrafish. The mineralization area and integrated optical density (IOD) of skull alizarin red staining were analyzed using Image-Pro Plus (IPP, Media Cybernetics, United States).

### Statistical Analysis

The data were expressed as mean ± standard deviations (SD). Statistical analysis was conducted using SPSS 19.0. A one-way analysis of variance (ANOVA) was used for multiple comparisons in the statistical analysis and a value of *p* < 0.05 was considered statistically significant.

## Results

### Kaempferol Promoted Cell Proliferation and Ameliorated the Proliferation Inhibition of MC3T3-E1 Cells Induced by DEX

**FIGURE 1:**
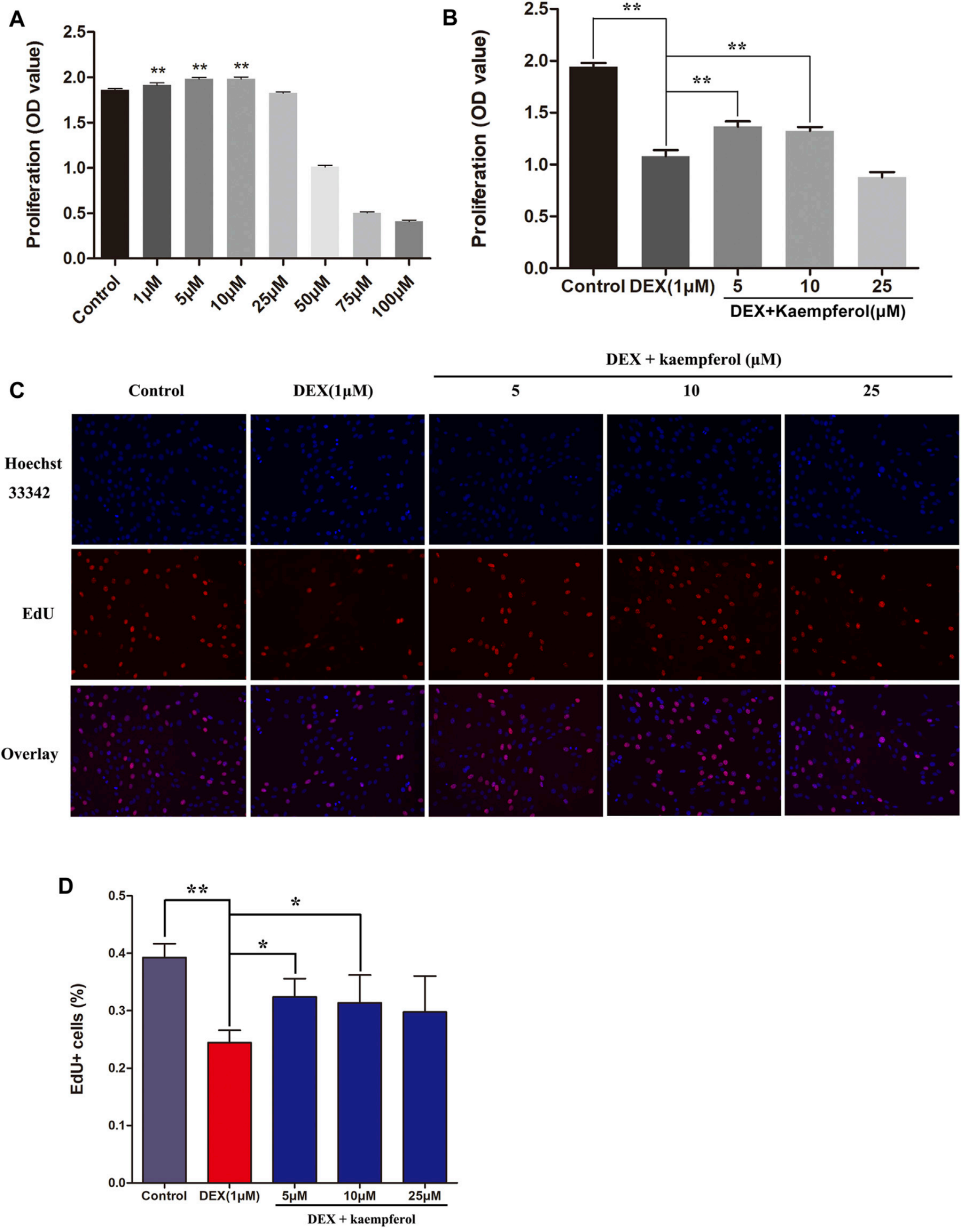
The effect of kaempferol on cell proliferation in DEX-treated MC3T3-E1 cells. **(A)** Cells were co-cultured with kaempferol (1–100 μM) for 3 days. **(B)** Cells were co-cultured with kaempferol (5, 10, and 25 μM) and DEX (1 μM) for 3 days. **(C, D)** Cells were treated for 24 h with kaempferol (5, 10, and 25 μM) and DEX (1 μM) and analyzed by EdU assay. *p < 0.05, **p < 0.01.

To explore the roles of kaempferol on the proliferation of MC3T3-E1 cells, CCK-8 assay and EdU assay were employed. Results indicated that kaempferol (1–10 μM) significantly promoted cell proliferation. However, kaempferol at the concentrations of 50–100 μM inhibited the proliferation of MC3T3-E1 cells (*p* < 0.01) (Figure 1A). Co-incubation of MC3T3-E1 cells with DEX for 3 days greatly inhibited the proliferation (*p* < 0.01). However, kaempferol (5 and 10 μM) ameliorated the inhibitory effect induced by DEX (*p* < 0.01) (Figure 1B). The EdU assay demonstrated that DEX significantly inhibited cell proliferation (*p* < 0.01), and the kaempferol (5 and 10 μM) ameliorated the inhibition of cell proliferation caused by DEX (*p* < 0.05) (Figures 1C,D).

### Kaempferol Eased Cell Cycle Arrest Induced by DEX in MC3T3-E1 Cells

**FIGURE 2:**
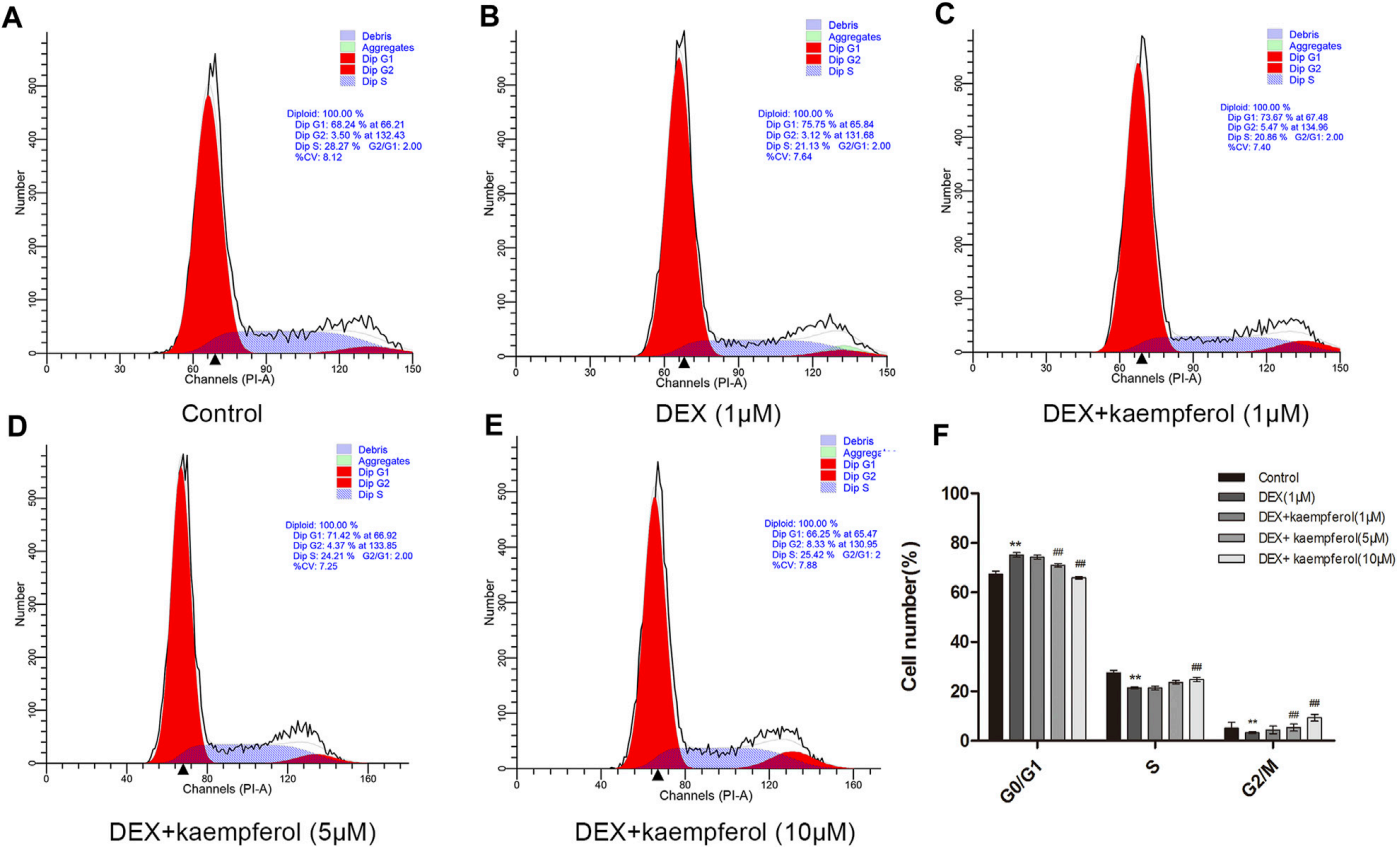
Kaempferol alleviated cell cycle arrest induced by DEX in MC3T3-E1 cells. Cells were co-cultured with kaempferol (1, 5, and 10 μM) and DEX (1 μM) for 3 days. A: Control; B: DEX (1 μM); C: DEX + kaempferol (1 μM); D: DEX + kaempferol (5 μM); E: DEX + kaempferol (10 μM); F: the bar graph of cell number (%) in cell cycles. **p < 0.01 vs. Control; ^##^ *p* < 0.01 vs. DEX.

Cells were co-cultured with kaempferol (1, 5, and 10 μM) and DEX (1 μM) for 3 days. The results indicated that DEX caused cell cycle arrest in the phase of G0/G1 (*p* < 0.01). However, kaempferol (5 and 10 μM) eased cell cycle arrest induced by DEX in MC3T3-E1 cells (Figure 2).

### Kaempferol Eased Cell Apoptosis Induced by DEX in MC3T3-E1 Cells

**FIGURE 3:**
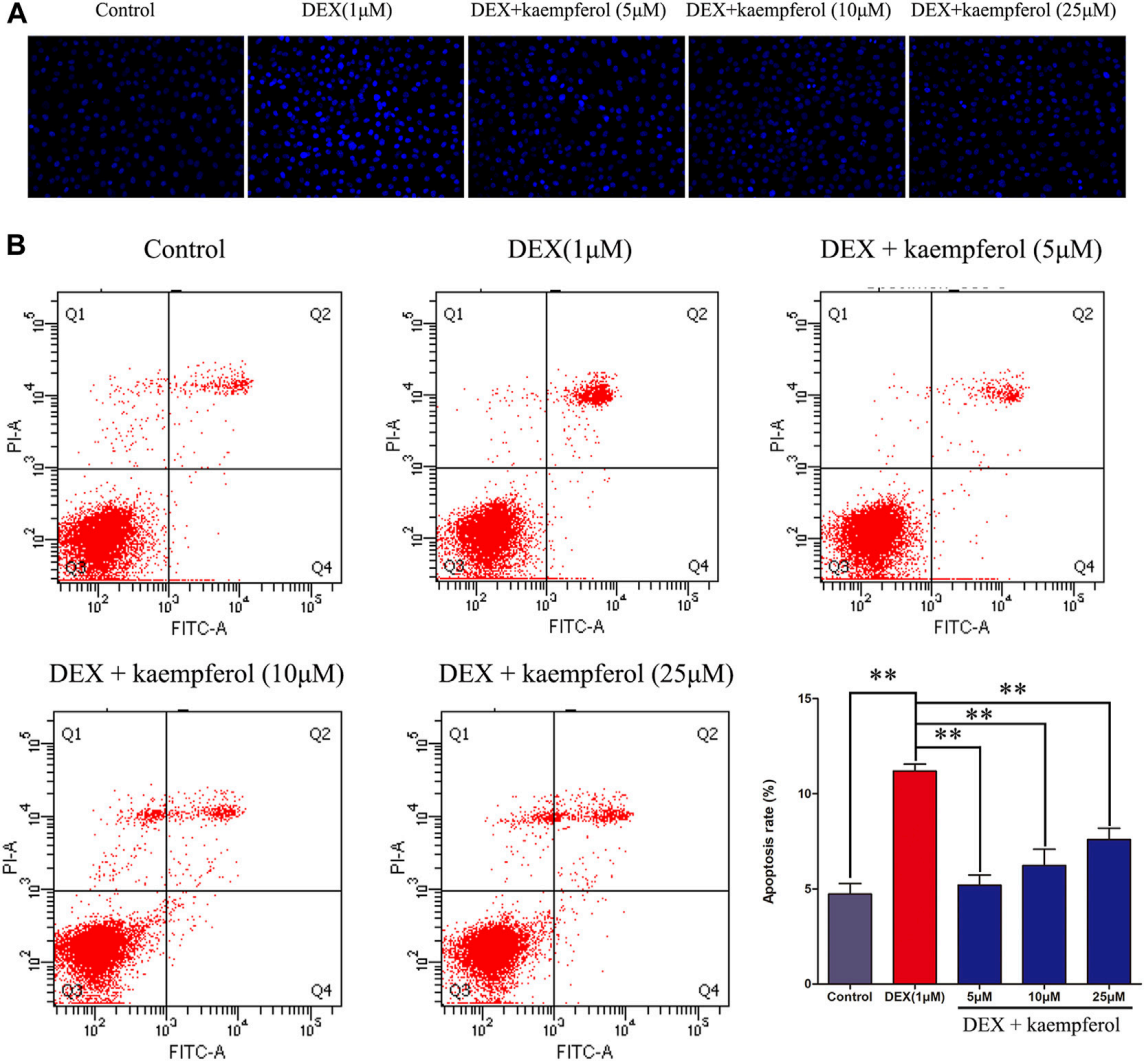
Kaempferol alleviated cell apoptosis induced by DEX in MC3T3-E1 cells. Cells were co-cultured with kaempferol (5, 10, and 25 μM) and DEX (1 μM) for 3 days. **(A)** The cells were stained with Hoechst 33342 and observed under fluorescence microscope; **(B)** Cells were stained with PI and annexin V-FITC to evaluate apoptosis by flow cytometry. *p < 0.05, **p < 0.01.

Cells were co-cultured with kaempferol (5, 10, and 25 μM) and DEX (1 μM) for 3 days. The results indicated that DEX caused cell apoptosis (*p* < 0.01). However, kaempferol (5, 10, and 25 μM) eased cell apoptosis induced by DEX in MC3T3-E1 cells (Figure 3).

### Kaempferol Attenuated Lessening of Cyclin D1 and Bcl-2 Expression and Increase of p53 and Bax Expression Induced by DEX

**FIGURE 4:**
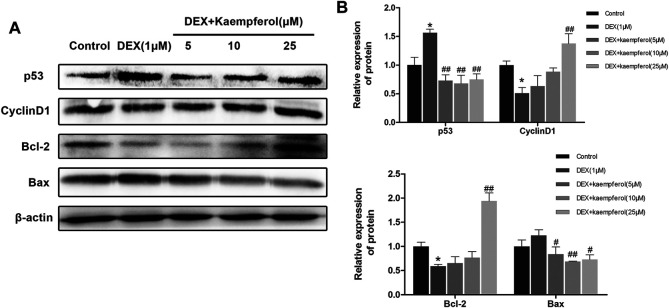
Kaempferol attenuated DEX-induced decrease of cyclin D1 and Bcl-2 expression and augment of p53 and Bax expression. **(A)** The proteins were detected with specific antibodies against p53, cyclin D1, Bcl-2, and Bax. β-actin was used as the loading control. **(B)** The quantification of p53, cyclin D1, Bcl-2 and Bax was indicated. *p < 0.05 vs. Control; ^#^ *p* < 0.05, ^##^ *p* < 0.01 vs. DEX.

MC3T3-E1 cells were co-cultured with DEX and kaempferol (5, 10, and 25 μM) for 3 days. The results showed that DEX decreased the protein expression of cyclin D1 and Bcl-2 and increased that of p53 and Bax. However, kaempferol reversed the effect of DEX, as indicated by the increased protein expression of cyclin D1 and Bcl-2 and the decreased expression of p53 and Bax in DEX-treated MC3T3-E1 cells (Figure 4).

### Kaempferol Increased the ALP Activity in MC3T3-E1 Cells Treated with DEX

**FIGURE 5:**
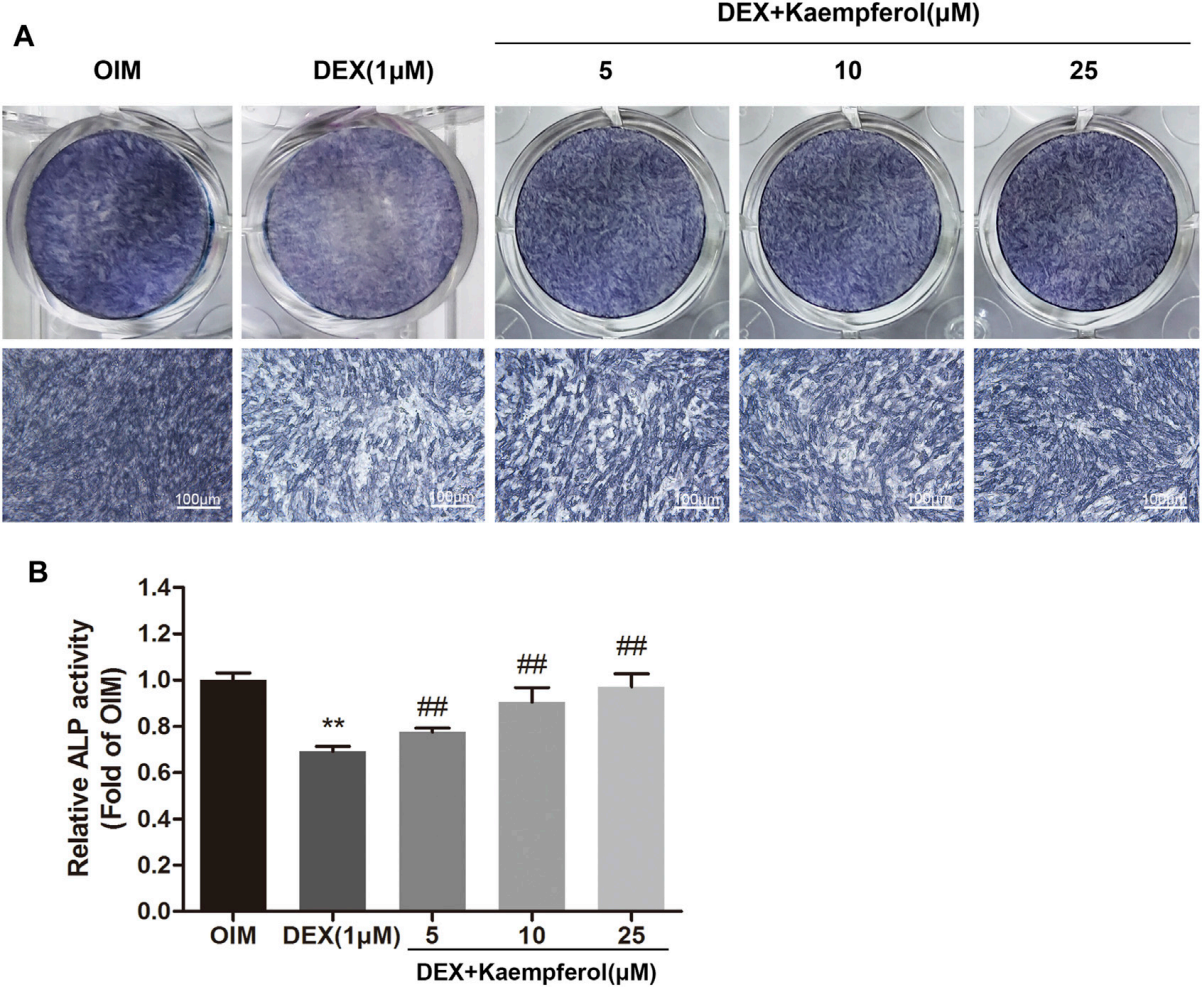
The effect of kaempferol on the ALP activity in DEX-treated MC3T3-E1 cells. **(A)** BCIP/NBT staining was conducted. **(B)** The ALP activity was determined after 5-days co-treatment of MC3T3-E1 cells with kaempferol (5, 10, and 25 μM) and DEX (1 μM) in OIM. **p < 0.01 vs. OIM; ^##^ *p* < 0.01 vs. DEX.

Next, we evaluated whether kaempferol would increase the ALP activity in DEX-treated MC3T3-E1 cells. MC3T3-E1 cells were cultured in OIM and incubated with DEX (1 μM) and kaempferol (5, 10, and 25 μM) for 5 days. As a result, DEX significantly decreased the ALP activity in MC3T3-E1 cells. In contrast, kaempferol (5, 10, and 25 μM) significantly enhanced the ALP activity in DEX-treated MC3T3-E1 cells (*p* < 0.01) (Figure 5).

### Kaempferol Increased the Mineralization in DEX-Treated MC3T3-E1 Cells

**FIGURE 6:**
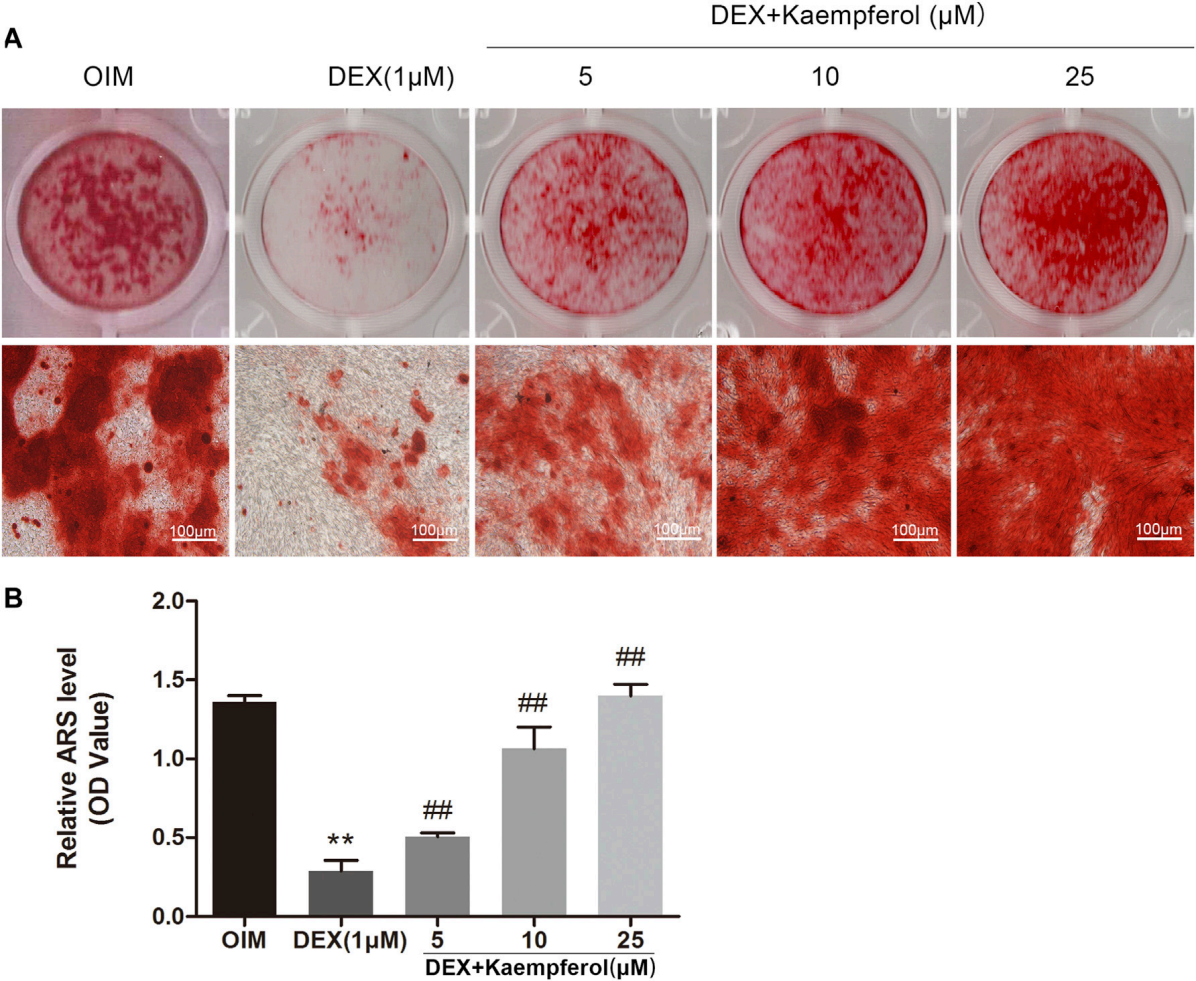
The effect of kaempferol on the mineralization of DEX-treated MC3T3-E1 cells. **(A)** Alizarin red S was used for staining on day 14. **(B)** The calcified nodules was quantified by extraction of alizarin red S with 10% cetylpyridinium chloride (CPC) on day 14. **p < 0.01 vs. OIM; ^##^ *p* < 0.01 vs. DEX.

MC3T3-E1 cells were cultured in OIM and incubated with DEX (1 μM) and kaempferol (5, 10 and 25 μM) for 2 weeks. Alizarin red staining was used to visualize the calcified nodules. DEX decayed the calcified nodules formation in MC3T3-E1 cells. In contrast, kaempferol (5, 10, and 25 μM) significantly promoted the calcified nodules formation, which was indicted by the quantitative data (Figure 6).

### Kaempferol Reversed the Inhibitory Effect of DEX on the Expression of Runx2 and Osx in MC3T3-E1 Cells

**FIGURE 7:**
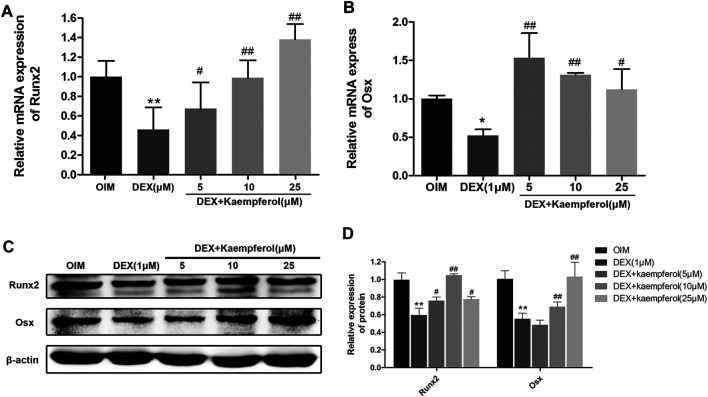
The effect of kaempferol on Runx2 and Osx expression in DEX-treated MC3T3-E1 cells. The mRNA expression of Runx2 **(A)** and Osx **(B)** were analyzed by qRT-PCR. **(C)** The expression of Runx2 and Osx were detected by western blot. **(D)** The quantification of Runx2 and Osx was indicated. *p < 0.01, **p < 0.01 vs. OIM; ^#^ *p* < 0.05, ^##^ *p* < 0.01 vs. DEX.

MC3T3-E1 cells were co-incubated with DEX (1 μM) and kaempferol (5, 10, and 25 μM) for 3 days. The results indicated that the mRNA levels of Runx2 and Osx in DEX + kaempferol group were significantly increased compared with DEX group (Figures 7A,B). MC3T3-E1 cells were co-incubated with DEX (1 μM) and kaempferol (5, 10, and 25 μM) for 5 days. The results from western blot analysis showed that expressions of Runx2 and Osx were significantly decreased in DEX (1 μM) group, which were reverse by co-incubation with kaemoferol (Figures 7C,D).

### Kaempferol Activated p38-MAPK and JNK MAPK Pathways in DEX-Treated MC3T3-E1 Cells

**FIGURE 8:**
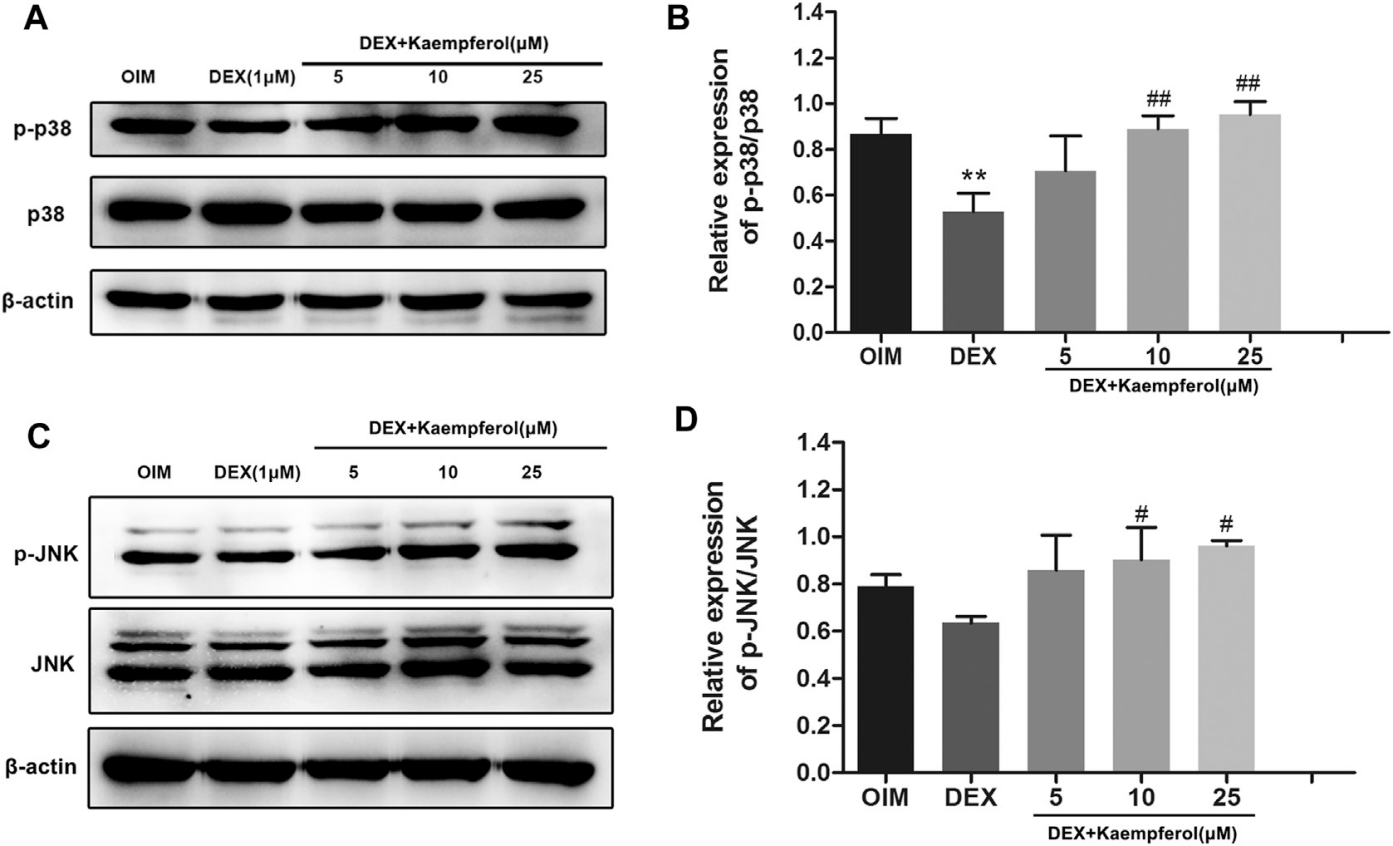
The effect of kaempferol on the phosphorylated protein of p-p38 and p-JNK in DEX-treated MC3T3-E1 cells. The phosphorylation levels of p38 **(A)** and JNK **(C)** were detected by western blot. The quantification of p-p38/p38 **(B)** and p-JNK/JNK **(D)** was calculated. **p < 0.01 vs. OIM; ^#^ *p* < 0.05, ^##^ *p* < 0.01 vs. DEX.

The phosphorylation levels of p38 and JNK in DEX-treated MC3T3-E1 cells were measured. The results indicated that DEX decreased p38 phosphorylation, and kaempferol restored the level of phosphorylated p38 (p-p38). DEX did not significantly change the level of phosphorylated JNK (p-JNK). In contrast, kaempferol promoted JNK phosphorylation in DEX-treated MC3T3-E1 cells (Figure 8).

### SB203580 and SP600125 Inhibited the Protective Effect of Kaempferol in DEX-Treated MC3T3-E1 Cells

**FIGURE 9:**
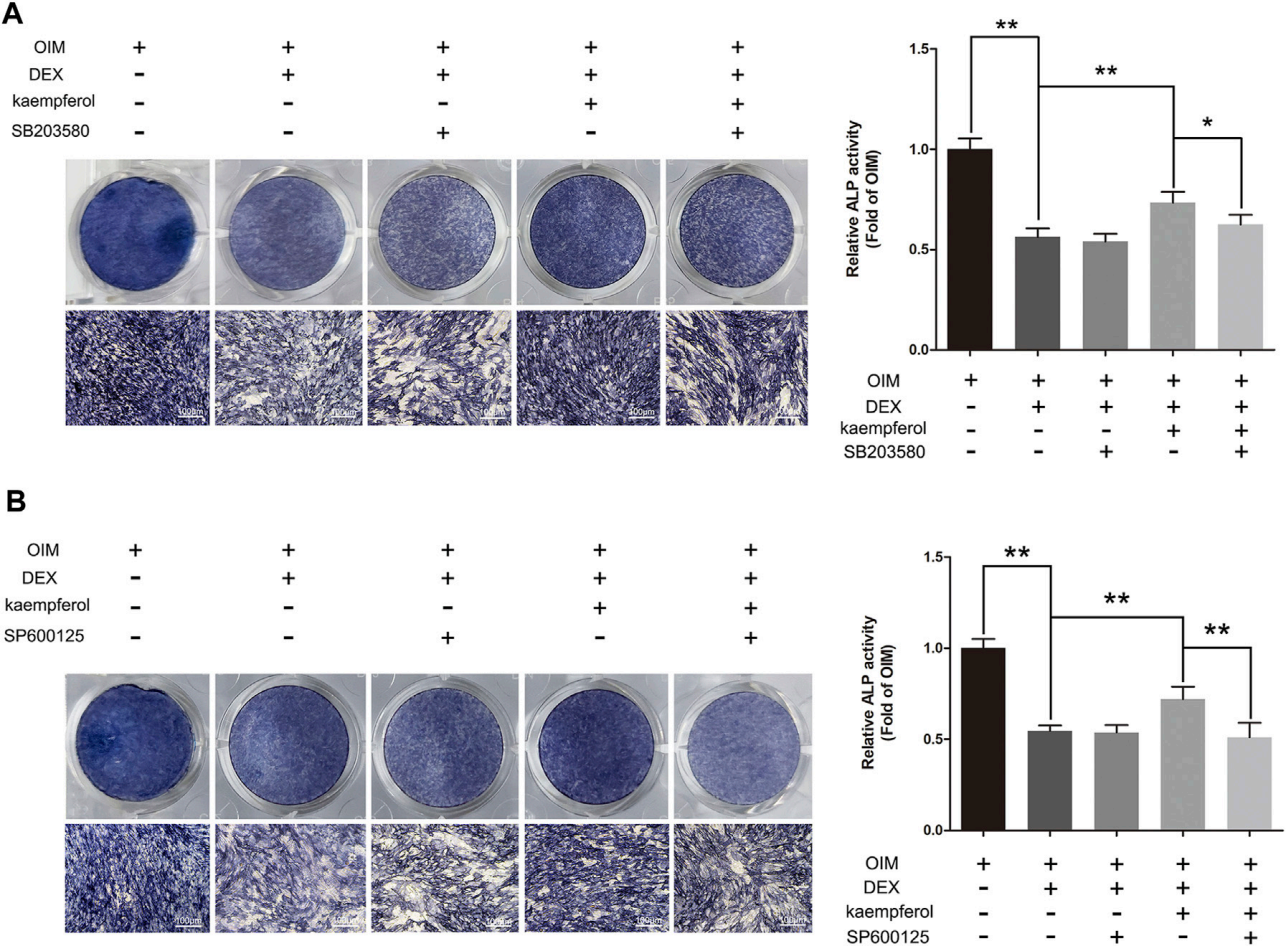
SB203580 and SP600125 inhibited the protective effect of kaempferol in DEX-treated MC3T3-E1 Cells. **(A)** ALP staining was performed with BCIP/NBT kit after the MC3T3-E1 cells were treated with SB203580, DEX and kaempferol for 5 days. **(B)** ALP staining was performed with BCIP/NBT kit after the MC3T3-E1 cells were treated with SP600125, DEX and kaempferol for 5 days. OIM, osteogenic induction medium; DEX, dexamethasone. *p < 0.05, **p < 0.01.

To further elucidate the role of p38-MAPK and JNK MAPK pathways in the protective effect of kaempferol, cells were treated with SB203580 (p38-MAPK inhibitor), SP600125 (JNK inhibitor), DEX and kaempferol. As shown in Figure 9, kaempferol (10 μM) significantly enhanced the ALP activity in DEX-treated MC3T3-E1 cells (*p* < 0.01). However, the addition of SB203580 or SP600125 significantly inhibited the ALP activity enhanced by kaempferol in DEX-treated MC3T3-E1 cells (*p* < 0.05).

### Kaempferol Attenuated DEX-Induced Decrease of Skull Mineralization in Zebrafish

**FIGURE 10:**
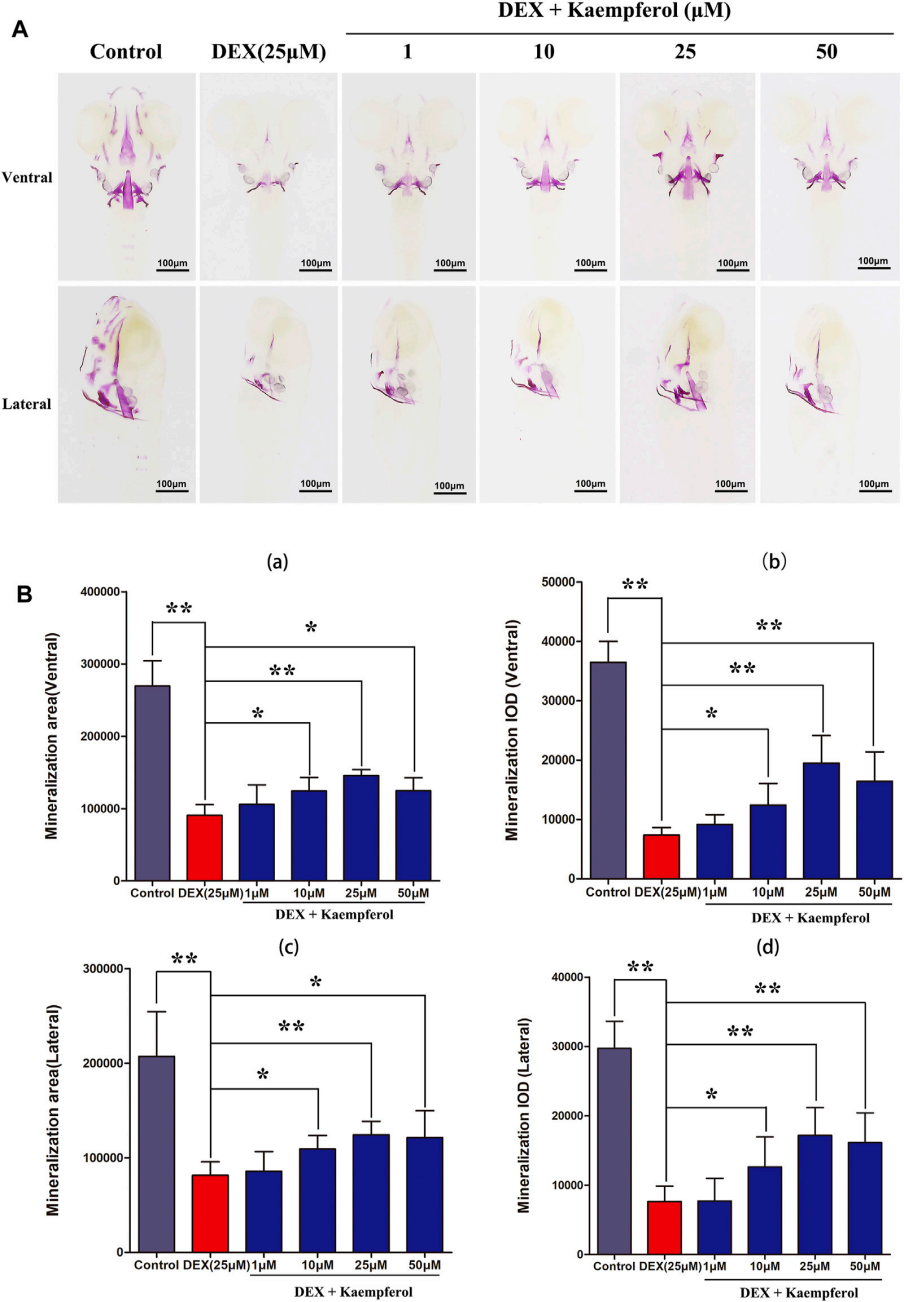
kaempferol attenuated DEX-induced lessen of skull mineralization in zebrafish larvae. **(A)** Zebrafish larvae in control group, DEX group and DEX + kaempferol (1,10, 25 and 50 μM)) group were stained with alizarin red to evaluate the degree of skull mineralization. **(B)** The area and IOD of skull alizarin red staining were analyzed by IPP. **(a)** ventral area of skull mineralization, **(b)** ventral skull mineralization of IOD, **(c)** lateral area of skull mineralization, **(d)** lateral skull mineralization of IOD. *p < 0.05, **p < 0.01.

Bone mineralization is a crucial indicator of bone formation. We observed the bone formation of zebrafish by staining with alizarin red. According to alizarin red staining area and IOD, the degree of skull mineralization can be determined. The results showed that bone mineralization area and IOD of skull in DEX group were obviously inhibited. However, the addition of kaempferol (10, 25, and 50 μM) attenuated the effect of DEX on decrease of bone mineralization area and IOD of skull in zebrafish (Figure 10).

## Discussion

Clinically, glucocorticoids are frequently used to treat non-infectious inflammatory diseases, such as asthma, inflammatory bowel disease and severe autoimmune diseases. It is well known that GIOP is a side effect, due to long-term administration of glucocorticoids. The mechanism might be associated with the pharmacological effect on bone by glucocorticoids, which inhibits bone formation and accelerates bone resorption (Jing et al., 2019). The proliferation and differentiation activities of osteoblasts are crucial to bone formation (Taipaleenmaki et al., 2012; An et al., 2016). In this study, the effect of kaempferol on proliferation and differentiation in MC3T3-E1 treated by DEX were explored.

We found that DEX attenuated cell proliferation and kaempferol dose-dependently ameliorated the effect of DEX in MC3T3-E1 cells. Cells proliferate for tissue renewal and repair damaged areas during wound healing. The alternations of cell cycle checkpoints are often seen in cancer cells, and regulation of the expression of cell cycle regulators governs the fate of cells (Urrego et al., 2014; Hardwick et al., 2015). In this study, DEX caused the cell cycle arrest in the phase of G0/G1 and kaempferol protected against the effect of DEX. This suggested that the effect of kaempferol on ameliorating DEX-induced proliferation inhibition is associated with cell cycle regulation.

P53 is considered as a guardian in the genome. When cellular DNA in the body was damaged, the p53 signaling pathway mediates repair and subsequent growth arrest. DEX can induce osteoblast cell death through activating glucocorticoid receptor-p53 signaling (Li et al., 2012; Zhen et al., 2014). Cyclin D1 is an important factor regulating the proliferation of various cells, which is negatively related to p53 (Sato et al., 2008). Bcl-2 is the main target molecule for the study of molecular mechanism of apoptosis. Bcl-2 can inhibit cell death caused by a variety of cytotoxic factors. Overexpression of Bcl-2 can enhance cell resistance to most cytotoxins. At present, the mechanism of Bcl-2 anti-apoptosis is mainly through antagonizing the pro-apoptotic gene Bax, inhibiting the release of pro-apoptotic cytochrome c from mitochondria into the cytoplasm and preventing cytochrome c in the cytoplasm from activating caspase. The Bax gene belongs to the Bcl-2 gene family, and the encoded Bax protein can form a heterodimer with Bcl-2 and inhibit Bcl-2. Studies have found that the ratio of Bax/Bcl-2 is a key factor in determining the strength of the inhibitory effect on apoptosis, and Bax is recognized as one of the most important pro-apoptotic genes. In this study, we found that DEX effectively promoted the expression of p53 and Bax and down regulated that of cyclin D1 and Bcl-2. Kaempferol alleviated DEX-induced cell cycle arrest and apoptosis, decrease of Bcl-2 and cyclin D1 expression, and up-regulation of Bax and p53 expression. Therefore, kaempferol attenuated the effect of DEX on proliferation inhibition and apoptosis by regulating p53, Bax, cyclin D1 and Bcl-2.

ALP is a marker of early osteogenesis and plays a critical role in bone formation (Harrison et al., 1995). Mineralization occurs in the last stage of osteogenic differentiation and is one of the common indicators of the late osteogenic differentiation. When the osteoblasts are cultured in the osteogenic induction solution for 1 week, calcified nodules will appear and gradually increase with the extension of the culture time. Alizarin red can chelate with calcium ions and form orange-red complexes in the mineralized nodules. Kaempferol could promote the formation of mineralization nodes (Kim, et al., 2016). In this study, we showed that DEX decreased the activity of ALP and the formation of calcified nodules in MC3T3-E1 cells. Kaempferol protected against the effect of DEX on MC3T3-E1 cells. These results revealed that kaempferol significantly attenuated the inhibition of DEX on osteogenic differentiation. A study showed that dexamethasone and kaempferol have a synergistic effect and promote cartilage differentiation synergistically (Gupta et al., 2021). However, we found that kaempferol protected against the effect of DEX on MC3T3-E1 cells. This discrepancy can be explained that 1) the different cell lines might exhibit distinctive responses to reagents, 2) the biological effects of dexamethasone are closely associated with its dosages, and 3) the biological situations in the different stages of cells exhibit specific pathways.

Runx2 acts as a transcription factor and plays a crucial regulatory role in the cellular proliferation and differentiation of osteoblasts (Komori, 2005; Wysokinski et al., 2015). Osx as an osteoblast-specific transcription factor can activate the expressions of an array of genes during cell differentiation (Sinha and Zhou, 2013). Runx2 increases the activity of the promoter of Osx through direct interactions with Runx2-binding element. Runx2 is the upstream controller of Osx (Sinha and Zhou, 2013). Our results found that DEX inhibited the mRNA and protein expression of Osx and Runx2. However, kaempferol reversed the effect of DEX. Collectively, kaempferol attenuated the inhibitory effect of osteogenic differentiation induced by DEX *via* mediating the expression of Runx2 and Osx.

MAPK signaling pathway is found to be closely correlated with cellular proliferation, differentiation, senescence, and apoptosis (Kim et al., 2014; Sun et al., 2015). Particularly, MAPK pathway exhibits a considerable role in osteoblast differentiation (Ma et al., 2015; Yu et al., 2016). Studies showed the flavonoids compounds, myricetin and puerarin, promoted osteogenic differentiation and mineralization *via* activating MAPK pathway (Fan et al., 2018; Yang et al., 2018). Our previous studies also showed that amentoflavone and apigenin improved the osteogenesis of MSC through up-regulating the activity of p38-MAPK and JNK pathways (Zhang et al., 2015; Zha et al., 2016). Here, we found that DEX down-regulated the expression of p-p38. However, kaempferol significantly up-regulated the phosphorylated protein of p-p38 and p-JNK, and it indicated that JNK and p38-MAPK pathways potentiated the biological effect of kaempferol. Additionally, JNK and p38-MAPK pathways have been shown to play crucial roles on regulation of protein expression of Osx and Runx2 (Wang et al., 2014; Wang et al., 2017). Therefore, kaempferol attenuated the suppressive effect of DEX on osteogenic differentiation by enhancing the expression of Osx and Runx2, which might be associated with p38-MAPK and JNK pathways.

The zebrafish has become a valuable disease model for osteoporosis research because its embryo is transparent and skeletal development is very similar to that of human (Carnovali et al., 2016). Our results showed that DEX decreased the bone mass and kaempferol significantly increased the bone mass in DEX-treated zebrafish, suggesting that kaempferol protected against GIOP.

## Conclusion

The present study demonstrated that kaempferol alleviated the inhibitory effect on osteogenic differentiation induced by DEX *via* activation of JNK and p38-MAPK pathways. Meanwhile, kaempferol alleviated DEX-induced inhibition of cyclin D1 and Bcl-2 *via* down-regulating p53 expression. Collectively, our study demonstrated that kaempferol could be a potential candidate for development as a new drug against GIOP.

## Data Availability

The raw data supporting the conclusions of this article will be made available by the authors, without undue reservation.
